# Leigh syndrome with developmental regression and ataxia due to a novel splicing variant in the *PMPCB* gene

**DOI:** 10.1038/s10038-024-01226-9

**Published:** 2024-02-19

**Authors:** Emma Matthews, Ella F. Whittle, Faraan Khan, Meriel McEntagart, Christopher J. Carroll

**Affiliations:** 1 Atkinson-Morley Neuromuscular Centre, Department of Neurology, St George’s University Hospitals NHS Foundation Trust, and Molecular and Clinical Sciences Research Institute, St George’s University of London, London, UK; 2 Genetics Section, Molecular and Clinical Sciences Research Institute, St. George’s, University of London, London, UK; 3 Department of Neuroradiology, St George’s University Hospitals NHS Foundation Trust, London, UK; 4 Medical Genetics, Clinical Developmental Sciences, St. George’s University of London, London, UK

**Keywords:** Neurological disorders, Genetics of the nervous system

## Abstract

Only five children with pathogenic *PMPCB* gene variants have been described and all carried missense variants. Clinical features included a Leigh-like syndrome of developmental regression, basal ganglia lesions and ataxia with or without dystonia and epilepsy. Three of the five died in childhood and none was older than age six when described. We report the first splice site variant in the *PMPCB* gene in a 39-year old individual who experienced developmental regression and ataxia following otitis media in childhood. A minigene assay confirms this variant results in aberrant splicing and skipping of exon 12.

## Introduction

To date only five individuals from four families with multiple mitochondrial dysfunctions syndrome 6 (MMD syndrome 6) due to bi-allelic variants in the *PMPCB* gene have been described [1]. Five different missense variants were identified, with the c.523C>T;(p.Arg175Cys) variant occurring in two unrelated children. The described phenotype included typical Leigh-like features of developmental regression and basal ganglia lesions, often associated with febrile illness, and with prominent cerebellar atrophy and increased T2 signal of the cerebellar cortex. Symptoms of dystonia, epilepsy and/or ataxia occurred in four. The onset of symptoms was by 12 months in each case and none of the children had obtained ambulation or speech. Three of the five were deceased by the age of six years. The surviving two were age five and six years at the time of the report. We describe to our knowledge the oldest patient to date with multiple mitochondrial dysfunctions syndrome 6 and the first carrying a *PMPCB* splicing variant.

## Materials and methods

All clinical procedures were conducted as part of routine clinical care. Written informed consent was obtained to publish clinical details. DNA from the proband and their parents was submitted to the UK Deciphering Developmental Disorder Study for analysis. Results from the proband were confirmed by bi-directional Sanger sequencing carried out in duplicate in an accredited NHS laboratory.

### Exon-trapping mini-gene assay

The pSPL3 splicing vector [2] was used in an exon-trapping mini-gene assay to confirm the suspicion of aberrant splicing caused by the variant c.1330-2A>T. *PMPCB* exon 12, harbouring either the reference base adenine at position c.1330-2 or the variant base thymine, was introduced to the pSPL3 plasmid via XhoI and BamHI restriction sites. The PMPCB exon is 76 base pairs (bp) with 149 bp of flanking intronic sequence upstream and 65 bp of flanking intronic sequence downstream included. Constructs were transfected into HEK293 cells via polyethylenimine (11460630, Fisher Scientific) at a 1:4 ratio and, after 24 h, total RNA was extracted, following manufacturer instructions, using TRIsure (BIO-38033, Bioline). RNA was converted to cDNA and amplified via a reverse-transcriptase polymerase chain reaction (PCR) using the Luna Universal One-Step RT-qPCR Kit (E3005, BioLabs) as per manufacturer instructions with the primers listed below. Resulting PCR product was separated on a 1.5% agarose gel for visualization.

pSPL3-F: 5ʹ-TCTGAGTCACCTGGACAACC-3ʹ

pSPL3-R: 5′-ATCTCAGTGGTATTTGTGAGC-3′

## Results

### Clinical case

**Fig. 1:**
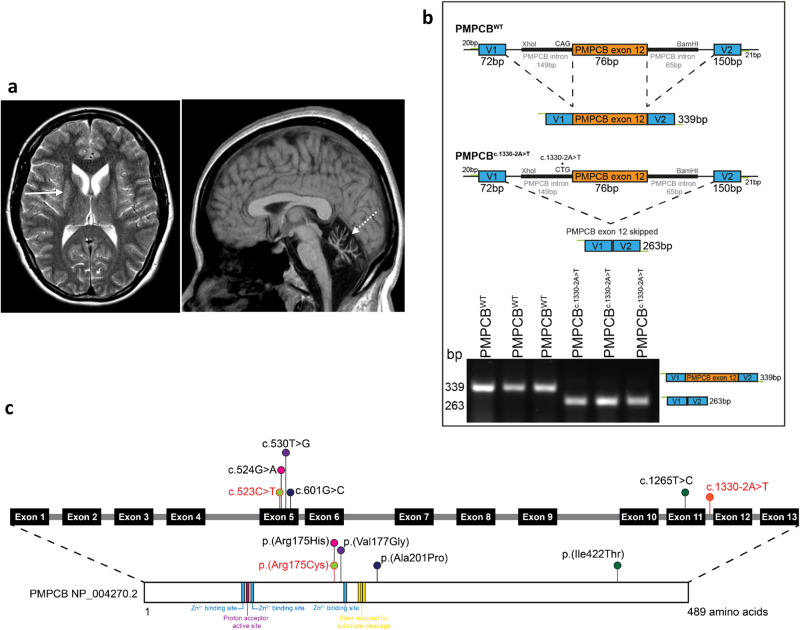
**a** MRI head images acquired at 39 years old. Axial T2 shows putamen atrophy and hyperintensity (arrow); Sagittal T1 shows cerebellar atrophy (dashed arrow). **b** Exon skipping resulting from the c.1330-2A>T variant demonstrated via an exon-trapping mini-gene assay. Top: Schematic showing the pSPL3 construct design for both wildtype and variant PMPCB exon 12. Bottom: Visualisation of mini-gene assay product showing a normal splicing product of 339 bp from the wildtype construct and a product of 263 bp produced by the construct carrying the c.1330-2A>T variant in transfected HEK293 cells. Three replicates are shown for each condition. This assay shows in vitro the resultant skipping of exon 12 by this variant. **c** Schematic of the *PMPCB* gene and protein, with all variants reported up to date indicated. Binding and activator sites indicated on the protein schematic derived from Uniprot: O75439. The variants identified in the individual in this study are shown in red

A 39 year old woman was born three weeks before term following an uneventful pregnancy. She was the only child of her parent’s union. Early history and development including speech was normal with walking achieved at 14 months. At the age of two years and five months she became quiet and lethargic. She was diagnosed with otitis media and subsequently admitted to hospital for five weeks with presumed encephalitis. She had regressed losing the ability to sit and speak and became incontinent having previously achieved continence. Medical records documented cerebellar atrophy on brain imaging. Over time she did regain speech but it was dysarthric. She regained the ability to sit and walk in a limited fashion but gait was very broad based and ataxic. She had multiple falls with injury e.g. broken teeth. From the age of six she has largely mobilised at home by moving on the floor due to severe ataxia. She attended a school for children with physical disabilities. She did learn to read and obtained some basic arithmetic skills but left school without completing any exams. Symptoms were relatively static until the age of 28 when she developed migraine headaches and severe dyspepsia with episodes of vomiting. Neurological examination demonstrated upper, lower limb and truncal ataxia with dysarthria, and nystagmus. MRI brain showed basal ganglia and cerebellar atrophy (Fig. 1a).

### Genetic results

The proband is compound heterozygous for the c.523C>T, p.(Arg175Cys);c.1330-2A>Tp.(?) variants in the PMPCB gene. Parental analysis confirmed the variants to be present in *trans*. Table 1 describes the predicted deleteriousness of the variants. Our exon trapping mini-gene assay indicates that the c.1330-2A>T variant causes aberrant splicing through the skipping of exon 12 (Fig. 1b).

**Table 1:** Variant information for the compound heterozygous variant presented in this study

cDNA impact	c.523C>T	c.1330-2A>T
RNA impact	r.(523C>U)	r.(1355_1430del)
Protein impact	p.(Arg175Cys)	p.(?)
RNA impact	–	r.1355_1430del
Transcript	NM_004279.3	NM_004279.3
Genome build	GRCh38	GRCh38
Chromosomal Location	7:103303907	7:103312054
gnomAD homozygotes	0	0
CADD Phred	28.3	35
SIFT prediction	Deleterious	NA
Polyphen	Probably damaging	NA
SpliceAI	Donor gain 0.01	Acceptor loss 0.99
REVEL	0.458	NA
ACMG	Pathogenic	Pathogenic

## Discussion

Leigh syndrome is the most common syndromic presentation of mitochondrial disease in early childhood [3]. Its clinical hallmark is developmental regression often following a minor illness with symmetrical basal ganglia lesions seen on brain imaging. Over 100 nuclear genes have been associated with this syndrome [4]. Our patient, like others reported with *PMPCB* variants, presented with Leigh like developmental regression and ataxia following a minor illness [1]. The onset of symptoms was later than previous cases and she had obtained both ambulation and speech in the first two years prior to illness. Although significantly impaired by ataxia after the onset of illness she made reasonable gains and has been relatively stable throughout adulthood, surviving to age 39 years. She did not develop the additional complications of dystonia or epilepsy described in several others. Her brain imaging revealed comparable changes of basal ganglia and marked cerebellar atrophy (Fig. 1a).

To our knowledge she is only the sixth person to be described with MMD syndrome 6 due to pathogenic variants in the *PMPCB* gene (Fig. 1c). She is heterozygous for the p.Arg175Cys missense variant. This has been seen in two other individuals, p.Arg175His present in a third, indicating this is a common recurring site for variation. In addition she is heterozygous for the first reported variant to affect splicing and cause skipping of exon 12 (Fig. 1b), c.1330-2A>T;p.(?).

The *PMPCB* gene codes for the catalytic β sub-unit of the mitochondrial processing protease (MPP). MPP consists of two subunits, α and β, encoded by separate genes and with a high degree of structural similarity. The MPP enzyme is key to ensuring the normal folding and maturation of mitochondrial proteins and, although the PMPCB protein holds the catalytic site, both subunits are required for proper processing [5]. The crystal structure of yeast MPP is known [6] and has been used to interpret pathogenicity of human *PMPCB* variants [1]. The known variant identified in this study, p.Arg175Cys, is a variant of a conserved residue in yeast and, when investigated using the yeast MPP crystal structure, was found to stabilise the protein fold through hydrogen bonds, bonds which cannot be maintained through the mutated cystine residue [1] (Supplementary Figure 1). The novel splicing variant identified in this study results in the loss of exon 12 in vitro (Fig. 1b). We predict that the resulting protein may not be functional, and that the milder phenotype observed in this individual compared to other reported cases could instead be due to variable aberrant splicing in vivo due to c.1330-2A>T, resulting in at least some residual normally spliced *PMPCB* mRNA present in tissues.

### Supplementary information

Supplementary Fig 1

Supplementary Fig 1 Caption
