# Supplementary figures and images for: Leigh syndrome with developmental regression and ataxia due to a novel splicing variant in the PMPCB gene
Source: J Hum Genet. 2024 Feb 19;69(6):283–5. doi: 10.1038/s10038-024-01226-9 (PMC11126369; doi:10.1038/s10038-024-01226-9)

## Slide 1
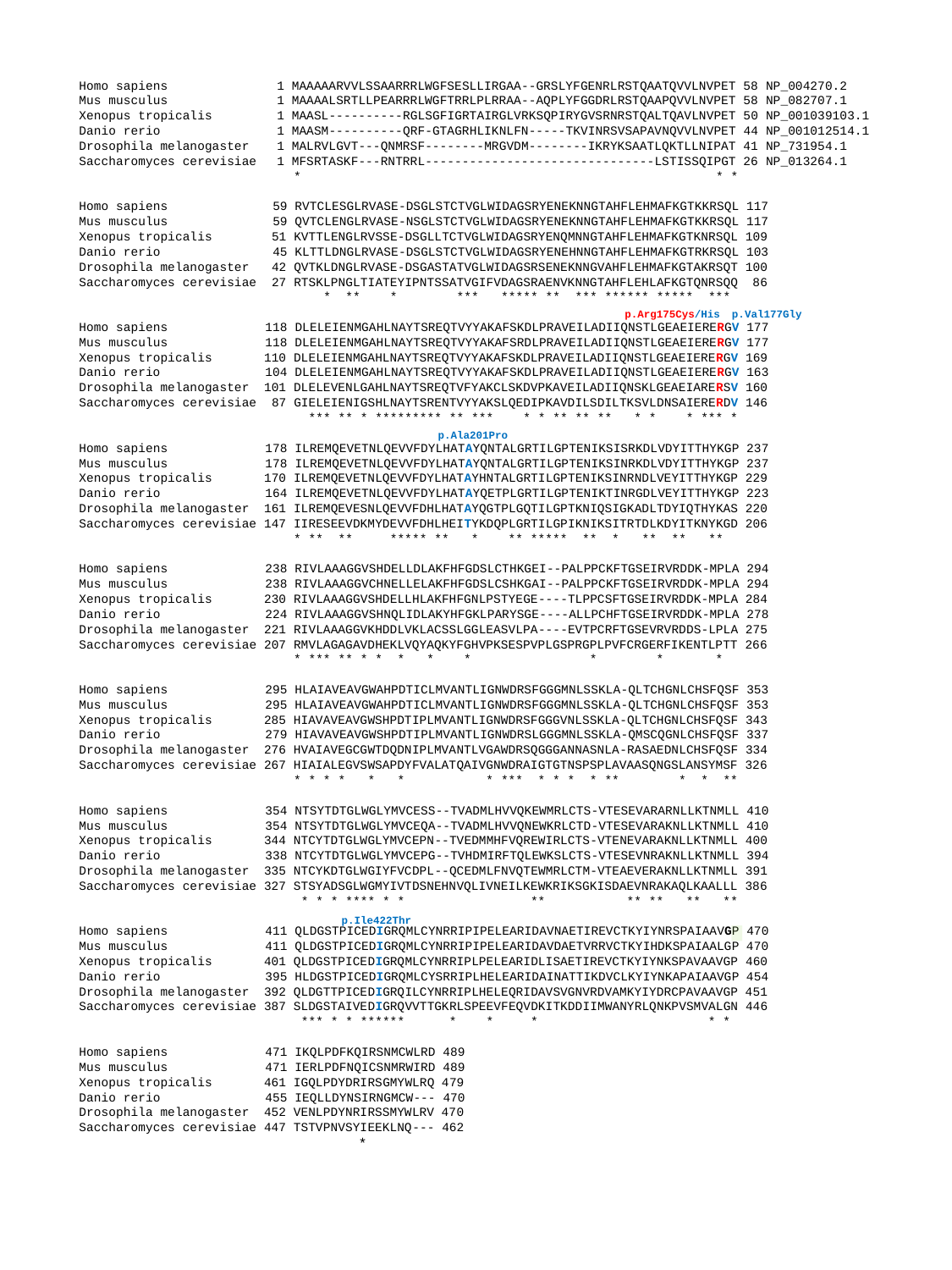

Supplement: Supplementary file 1 — Supplementary Fig 1 [file 10038_2024_1226_MOESM1_ESM.pptx]
