# Supplementary material for: Leigh syndrome with developmental regression and ataxia due to a novel splicing variant in the PMPCB gene
Source: J Hum Genet. 2024 Feb 19;69(6):283–5. doi: 10.1038/s10038-024-01226-9 (PMC11126369; doi:10.1038/s10038-024-01226-9)
Supplement: Supplementary file 2 — Supplementary Fig 1 Caption [file 10038_2024_1226_MOESM2_ESM.docx]

Supplementary Figure 1: Multiple alignment of the PMPCB protein across various species, with all known *PMPCB* variants reported to date indicated. The p.Arg175Cys missense variant identified in the individual in this study is shown in red. All other variants previously reported [1] are shown in blue.
